# Familial Progressive Hyperpigmentation: A Case Report

**DOI:** 10.1155/2012/840167

**Published:** 2012-04-18

**Authors:** Monica Yadav, Sugandha Ghonasgi, Rohit Shah, S. M. Meghana

**Affiliations:** ^1^Department of Oral Pathology and Microbiology, Terna Dental College and Hospital, Sector No. 22, Nerul 400706, India; ^2^Department of Periodontics, Terna Dental College and Hospital, Sector No. 22, Nerul 400706, India; ^3^Department of Oral Pathology, Terna Dental College and Hospital, Sector No. 22, Nerul 400706, India

## Abstract

Familial progressive hyperpigmentation (FPH) is a rare genodermatosis characterized by hyperpigmented patches in the skin and mucous membranes, present in early infancy, and increase in size and number with age. The genetic basis for FPH remains unknown. We report an unusual case of familial progressive hypermelanosis in a 17-year-old male patient with family history, who presented with a peculiar progressive oral pigmentation disorder. Diagnosis was confirmed by a series of hematological, biochemical, and histopathological investigations. Our paper stresses the need for the dentist to be aware of the systemic conditions that can also manifest in the oral cavity.

## 1. Introduction

Familial progressive hyperpigmentation (FPH) is a rare genodermatosis first reported by Chernosky et al. in 1971 [1]. Melanosis universalis hereditaria, melanosis diffusa congenita, universal acquired melanosis, and familial universal or diffuse melanosis are just some of the other terms coined by various authors to describe patients with a generalized diffuse hypermelanosis without systemic symptoms, but often with a familial pattern [2]. This condition has been described mostly in blacks, Hispanics, and oriental individuals, with both the sexes being affected equally [3]. Mode of inheritance is still debated upon since a number of inheritance patterns are recognised, mainly: (1) autosomal dominant (Chernosky et al. 1971 [1], Rebora and Parodi 1989 [4], Debao and Ting 1991 [5]); (2) autosomal recessive with germ-line mosaicism (Wende and Baukus 1919, Pegum 1955) [3].

Though such cases have been previously reported in dermatology, no such case has been reported in dental literature even though there are significant oral findings in these patients.

## 2. Case Report

**Figure 1:**
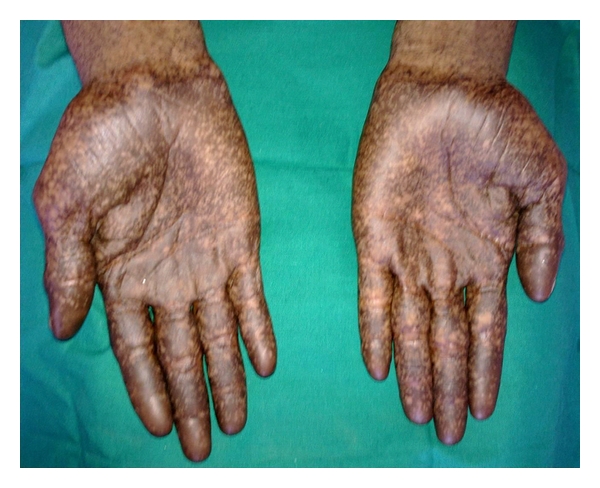
Photographs showing dark mottled pigmentation on the hands.

**Figure 2:**
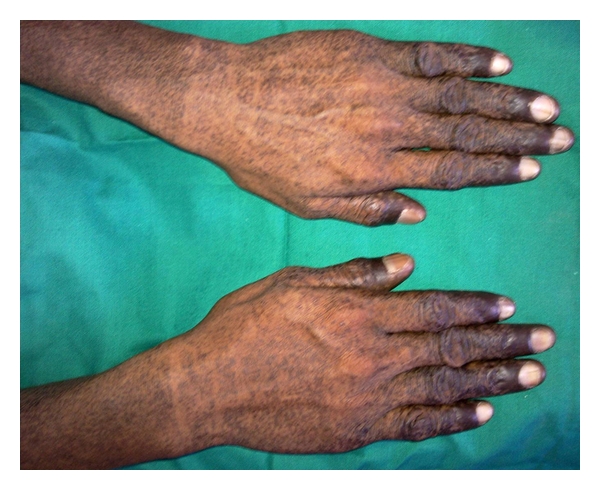
Photographs showing dark mottled pigmentation on the hands.

**Figure 3:**
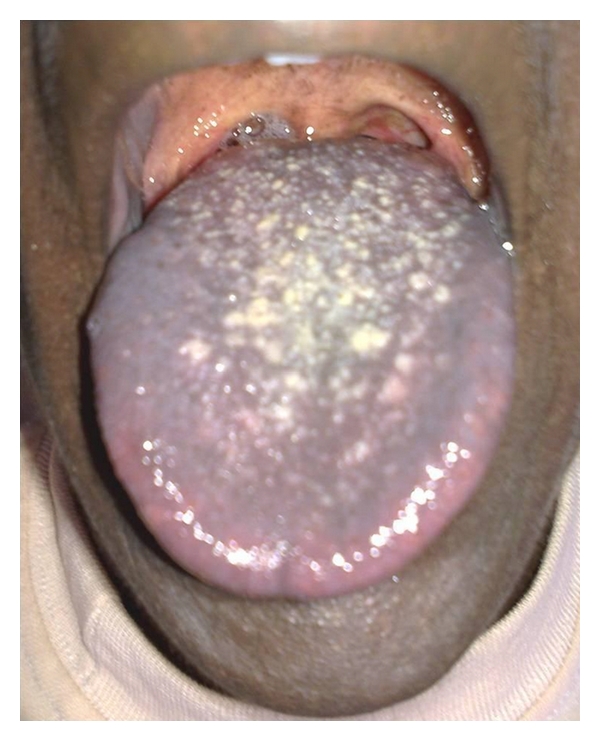
Generalised pigmentation seen on the dorsum of tongue with prominent lingual papillae.

**Figure 4:**
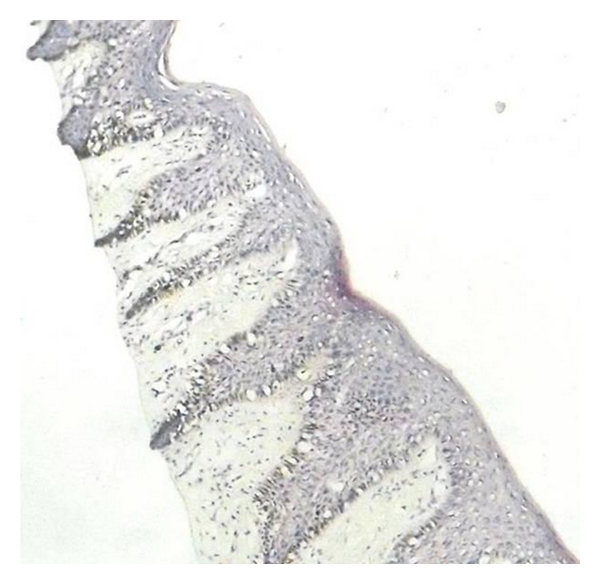
Increase in the number of melanocytes in the basal and suprabasal layers (Masson Fontana, 10×).

A 17-yr-old male patient reported to the department with a complaint of pain in the lower right side of the arch since 2 weeks. General examination revealed the presence of abnormal spotty hyperpigmentation all over his body. The pigmentation was more pronounced over the extremities (Figures 1 and 2). The pigmentation was present since birth and eventually increased thereafter. No other systemic abnormality was observed. He also reported that his brother and maternal uncle showed similar skin changes. Intraorally, generalized pigmentation was seen over the mucous membranes and observed to be concentrated on the tongue, palate, and gingiva. The lingual papillae were seen as prominent whitish spots, which on palpation appeared to be leathery in consistency (Figure 3). A grossly decayed 46 was found and advised for extraction, followed by prosthetic rehabilitation. A routine hematological investigation was done along with estimation of serum ACTH, *α* and *β* MSH, T_3_ T_4_ TSH, and Cortisol levels to ascertain the cause for the hyperpigmentation. Hematology and blood chemistry did not reveal any abnormalities. After an opinion from the dermatologist, an initial diagnosis of familial progressive hyperpigmentation (FPH) was made. The patient was then scheduled for an oral incisional biopsy under local anaesthesia. Light microscopic examination of H&E sections taken from the edge of the hyperpigmented areas displayed strong basilar and suprabasilar hyperpigmentation. Numerous melanophages seen as dark brown pigment in the subepithelial layers were also noted. Edge of the tissue from the apparently normal appearing gingiva showed only a slight basal hyperpigmentation but virtually no melanophages in the connective tissue. Further, presence of melanin in the section was confirmed by bleaching using hydrogen peroxide and to rule out the possibility of hemosiderin pigment. Also, Masson-Fontana stained sections showed an increase in the number of melanocytes in the basal and suprabasal cell layers (Figure 4).

This confirmed the provisional diagnosis of familial progressive hyperpigmentation (FPH).

## 3. Discussion

Familial progressive hyperpigmentation is a distinctive, dominantly inherited genodermatosis, characterized by patches of hyperpigmentation present at birth, which increase in size and number with age [6]. Clinical features have been described variously as intense pigmentation either as jet black, dark brown, or bronze. Patchy hyperpigmentation occurring at birth or early infancy progresses relentlessly until it encompasses the entire body. Initial site of involvement is either the face or groin. Various patterns of pigmentation have been described. Diffuse pigmentation due to coalescence of the macules or segmental patterns consisting of streaks/whorls located mainly on the torso (Chernosky et al., 1971) is the most commonly described pattern [1, 3]. Other areas to be commonly affected are palms, soles, lips, oral mucus membrane, and conjunctiva. On the other hand, hair remains unaffected. Occasionally, in adult life, there is a tendency to fade resulting in a bronze color or mottled appearance. Apart from pigmentary disorder, no other developmental anomalies are noted [2].

Oral mucus membrane is characterized by a mottled appearance [3]. Frequently involved oral sites are lips, buccal mucosa, tongue, and hard palate. Occasionally the pigmentation can obscure the lingual papillae on the dorsum of the tongue. Histologically, biopsy from the involved site shows a significant increase in melanin throughout the epithelium including being more pronounced in the basal layer. Epithelial melanocytes are normal in size and number, a finding that has been confirmed by DOPA stains [3, 7]. However, the connective tissue remains unaffected. Electron microscopic studies of skin biopsy have demonstrated the presence of melanin in all layers of epidermis. Melanocytes are seen to contain a large number of melanosomes that are normal in size, shape, and structure [3]. Immunohistochemical studies suggest an increased expression of hepatocyte growth factor (HGF), stem cell factor (SCF), and keratinocyte growth factor (KGF) in fibroblast-like cells of the upper dermis in hyperpigmented lesions of patients, compared to control healthy skin. Cardinali et al. suggest that a persistent activation of fibroblasts abnormally stimulating melanocyte functions is involved in hyperpigmentation disorders [8].

Hematological and biochemical investigations, including hormone (ACTH, MSH, T3, T4, and cortisol) levels are generally found normal in these subjects [3]. The common differential diagnoses a dentist should remember in this case are the following: Carbon Baby syndrome, Addison's disease, Cushing's disease, Melasma (chloasma), Peutz-Jeghers syndrome, exposure to certain chemicals such as salicylic acid, bleomycin, and mercury poisoning, smoker's melanosis, and haemochromatosis [9].

No known dental treatment is documented in previous literature. There is also no literature on the malignant potential of these lesions or any other pathological change. The treatment is based solely on cosmetic purposes. The cosmetic oral treatment including depigmentation procedure of the gingiva can be carried out. As this patient was not concerned about aesthetics, no such treatment was carried out. Since there is no literature available on the prognosis of these lesions, it is recommended to keep these lesions under periodic evaluation.
